# Recent neuroscience advances of interest to neurosurgeons, neurologists and neuroscientists — June 2010

**DOI:** 10.4103/2152-7806.65053

**Published:** 2010-07-01

**Authors:** James I Ausman

**Affiliations:** Editor-in-Chief, *Surgical Neurology International*, USA

## SYNTHETIC GENOME - SYNTHETIC LIFE?

In a landmark study reported in *Science Express* (www.sciencexpress.org/20 May 2010/Page 1/10.1126/science.1190719; Science News: Science; 328, P958, 2010), J. Craig Venter and colleagues reported the building of a genome (whole DNA sequence) from the start to make a DNA that produces synthetic life in a bacterium. It took 10 years of accumulated work to reach this stage. The scientific team built a synthetic copy of the DNA genome of a bacterium, M. *mycoides*. They assembled the sequences of the bases necessary to construct the synthetic DNA and then used a yeast cell to assemble sequences of 10,000 DNA bases and then the 100,000 sequences of bases into the complete genome. They then transferred the synthetic genome they made into another bacterium, M. *capricolum*. Initially, the cell did not divide because of a mistake in one of the DNA bases in the whole chain. When this base was corrected, the bacterium finally grew into a colony of bacteria. The colony grew like the M. *mycoides* from which the synthetic DNA was built. So, a different bacterial colony emerged being guided by the DNA from the original species. The work has been described as “a defining moment in the history of biology and biotechnology,” “a technological milestone,” “amazing accomplishment.” This has been called the artificial creation of life. At a minimum, it will lead to the production of many proteins on a large scale. Will it lead to the artificial production of life? The discovery will alter the course of medicine in the 21^st^ century.

## PATHWAYS FOR ADDICTION TO NICOTINE REVEALED

In the June 17, 2010, issue of the *New England Journal of Medicine*, Neal Benowitz in a paper entitled “Nicotine Addiction” reviewed the evidence to date for multifactorial causes and metabolic pathways involved in nicotine addiction. This is a wonderful summary of the many factors that lead to behavior change that we all witness worldwide in smokers. The causes relate to an interplay of learned or conditioned factors — environmental, social, genetic, metabolic; and the engineering and design of the cigarette to produce the addiction.

Cigarette smoke containing the nicotine is inhaled in the lungs and rapidly passes to the arterial system, which takes the nicotine to receptors in the brain. There are multiple receptors, which react to the nicotine in different ways, and these receptors are determined genetically. These nicotinic receptors open the cellular channels to calcium and sodium influx, leading to depolarization of the cell membranes and cell firing with release of transmitters at the nerve terminal. Dopamine is the transmitter principally released, which produces a pleasurable experience. Nicotine also stimulates the release of gama amino butyric acid (GABA), which inhibits dopamine release; however, over time, with smoking this receptor becomes desensitized to nicotine, and its inhibitory influence over dopamine is lost. Dopamine stimulates a reward system in the brain, which is probably primitive and which is located in the nucleus accumbens located in the anterior part of the third ventricle; and a nuclear group in the ventral tegmental region of the midbrain. The locus coeruleus, in the floor of the 4^th^ventricle at the obex, is also considered part of this reward system but is not mentioned in association with smoking. These nuclei are also associated with the limbic system. Monamine oxidases normally aid in the metabolism of dopamine, but cigarette smoke contains chemicals that inhibit the monamine oxidases, leading to the persistence of the transmitter dopamine and its effects.

With continued smoking and neural stimulation, neural plasticity leads to an increase in the number of nicotine receptors. Thus, when a person tries to stop smoking, the withdrawal symptoms occur stimulated by the production of corticotrophin-releasing factor (CRF), which leads to anxiety and other effects. To avoid these withdrawal effects, the individual will smoke taking in nicotine to occupy the receptors and produce a pleasure effect and the blockage of the withdrawal symptoms. If the CRF receptors in the brain are blocked, then the anxiety in withdrawal is not seen. Eventually, smoking produces a conditioned behavior that supports the pleasure and inhibits the withdrawal symptoms. Social situations in which there is stress can lead to smoking to avoid the stress with the resulting pleasurable response. Thus, the addiction takes hold. The dosing the smoker uses is dependent on his or her own metabolism of the nicotine. Those who metabolize the drug slowly need fewer cigarettes while those who metabolize the drug rapidly smoke more. Women have a higher metabolic rate of nicotine than men and smoke more cigarettes. Genetic inheritance can determine the type of nicotine receptors a person has. Studies on twins have shown a high degree of similarity in inheritance of the smoking habit, which is thought to be genetically based. Scientific work is being done on drugs to occupy the nicotine receptors as a means of preventing the addictive effects of nicotine. No doubt, these pathways are similar to the addiction pathways with other substances.

Smoking begins before the age of 20, with 20% to 25% of the young becoming addicted. Only 3% of people can overcome the addiction. This paper explains why 45 million people in the USA are smokers and 50% will die of complications of smoking. One million smokers die a year in China. So, smoking and nicotine addiction are huge health problems with social consequences.

This article is an excellent example of the interplay of genetics, social forces, body metabolism, neural plasticity; and the involvement of the limbic system, nucleus accumbens and the ventral tegmental region of the midbrain in drug toxicity. It also explains behavioral changes that are seen in people.

Now, imagine a smoker who commits a crime and whose lawyer defends his/ her action as one over which he has no control as he/ she cannot overcome the neuroplastic changes in their brain or the genetic changes that lead to the addiction and withdrawal symptoms leading the person to commit murder. So, as scientific knowledge of the actual functions of the human body increases, the social implications will become profound.

As neurosurgeons, we take out the metastasis from the lung cancer smoking produces, radiate it or treat it with chemotherapy. Some have vascular changes that complicate the outcomes of surgery. How many smokers who are hospitalized have withdrawal symptoms that we do not recognize?

## ANATOMICAL SITES THAT ARE AFFECTED IN TRANSIENT GLOBAL AMNESIA (TGA)

In the June 11, 2010, issue of *Science*; 328:1412-1425, Bartsch *et al*. studied 14 patients presenting with pure TGA without any focal neurological signs and amnesia resolving in 24 hours and compared them with healthy controls. A detailed battery of psychological and memory tests were performed on the patients 4 hours after onset and 14 days later. Multiple-sequence brain magnetic resonance (MR) scans on a 3 Tesla magnetic resonance scanner (3T-MRI) were imaged 24 to 72 hours after onset, when the lesions would be most highly detected. The spatial memory, or the ability to navigate in space with memory of surroundings, was impaired in all the TGA patients. MR imaging showed consistent lesions in the CA1 (cornu ammonis 1) sector of the hippocampus. The lesions were distributed on an anterior-posterior axis along the hippocampus, all in the CA1 sector. The size of the lesions and duration of the TGA correlated with the impairment in memory performance. Focal MR spectroscopy of the lesions indicated elevated lactate levels, suggesting anaerobic metabolism in response to the ischemic stress. The study indicates the focal lesion in pure TGA produces selective involvement of the CA1 sector of the hippocampus, producing defects in spatial memory and learning.

## A MOLECULE PROMOTING POSITIVE GROUP BEHAVIOR

In the same issue of *Science*; 328:1408-1411, 2010, De Dreu *et al*. studied the effects of oxytocin on human behavior. In controlled experiments with one group inhaling a placebo and the other oxytocin, those who inhaled oxytocin behaved more altruistically, or with selflessness and concern for others. Yet when the individuals in the group that inhaled oxytocin perceived that they were threatened, they bonded together defensively against the other group. The placebo-treated group demonstrated no such behavior. Others have found that genetic differences in the oxytocin receptor will produce different empathetic responses. Those with the pure gene will demonstrate more empathy than those with only one copy of the gene (Rodrigues *et al*., Proc Nat Acad Sci 106:21437-41, 2009). The oxytocin molecule produces a bonding effect between mother and infant and appears to have a similar role in adult behavior. This is an additional paper indicating the relationship between the molecular, neural networked brain and behavior. Does another molecule produce bonded aggressive behavior that one sees in gangs, militants or armies? What will be the implications of such a discovery? Like the previous two reports, this study also shows that the artificial boundaries between psychiatry, psychology, neurology, neuroradiology and neurosurgery will disappear in the 21^st^ century as we all discover we are working on the same brain from different aspects.

